# Vegetarian Diets along with Regular Exercise: Impact on High-Density Lipoprotein Cholesterol Levels among Taiwanese Adults

**DOI:** 10.3390/medicina56020074

**Published:** 2020-02-13

**Authors:** Shu-Lin Chang, Kuan-Jung Lee, Oswald Ndi Nfor, Pei-Hsin Chen, Wen-Yu Lu, Chien Chang Ho, Chia-Chi Lung, Ming-Chih Chou, Yung-Po Liaw

**Affiliations:** 1 Institute of Medicine, Chung Shan Medical University, Taichung City 40201, Taiwan; sherry.chang@kimo.com; 2 Department of Public Health and Institute of Public Health, Chung Shan Medical University, Taichung City 40201, Taiwan; jasminemachi@gmail.com (K.-J.L.); nforoswald2@yahoo.com (O.N.N.); c0701chen@gmail.com (P.-H.C.); yvonne841026@gmail.com (W.-Y.L.); dinoljc@csmu.edu.tw (C.-C.L.); 3 Department of Physical Education, Fu-Jen Catholic University, New Taipei City 24205, Taiwan; 000969@mail.fju.edu.tw; 4 Research and Development Center for Physical Education, Health and Information Technology, Fu-Jen Catholic University, New Taipei City 24205, Taiwan; 5 Department of Medical Imaging, Chung Shan Medical University Hospital, Taichung City 40201, Taiwan

**Keywords:** HDL-C, physical exercise, cardiovascular disease

## Abstract

*Background and objectives:* High-density lipoprotein cholesterol (HDL-C) is important for improving risk estimates of atherosclerotic cardiovascular disease. We investigated the effect of omnivore and diverse vegetarian diets in connection with exercise on HDL-C. *Materials and Methods:* Historical data of 9588 biobank participants (4025 exercisers and 5563 non-exercisers) aged 30–70 years were categorized as omnivores (n = 8589), former vegetarians (n = 544), lacto-ovo vegetarians (n = 417), and strict vegetarians (n = 38). We used multiple linear regression for analyses. *Results:* HDL-C levels were higher in exercisers compared to non-exercisers. Compared with omnivores, strict vegetarians had decreased levels of HDL-C (*β* = −5.705; *p* = 0.001) followed by lacto-ovo vegetarians (*β* = −3.900; *p* < 0.001) and former vegetarians (*β* = −0.329; *p* = 0.475). The test for trend was significant (*p* < 0.001). After categorization by exercise modalities, the *β*-value was −13.984 for strict vegetarians, −4.419 for lacto-ovo vegetarians, and −1.864 for former vegetarians, respectively (*p* < 0.05). There was an interaction between diet and exercise (*p* = 0.009). Omnivores who exercised regularly had significantly higher HDL-C, whereas strict vegetarians who exercised regularly had significantly lower HDL-C. *Conclusions:* In summary, strict vegetarian diets in conjunction with regular exercise might not serve as healthful behaviors to be implemented in everyday life considering the negative impact on HDL-C.

## 1. Introduction

Dyslipidemia (defined as high total cholesterol, high low-density lipoprotein (LDL) cholesterol, or low high-density lipoprotein (HDL) cholesterol) is one of the modifiable risk factors linked to cardiovascular disease (CVD) [1]. It is predominantly characterized by low concentrations of HDL cholesterol in Asia and the Middle East [2]. Low HDL cholesterol is a lipid fraction that serves as a marker for poor metabolic health [3] and may occur in the presence or absence (i.e., isolated HDL-C) of other lipoprotein abnormalities [4,5]. A 1 mg/dL HDL reduction has been associated with a 3–4% increase in the prevalence of coronary artery disease [6,7].

Physical exercise and a healthful diet have been independently associated with lower rates of cardiovascular disease morbidity and mortality [8]. Regular exercise is one of the ways to increase HDL cholesterol levels [9]. According to a study previously conducted in Japan, an exercise of at least three to four times per week for at least 20 min would help increase HDL levels [10]. In that study, the authors found that each additional 10 min increase in exercise duration yielded an additional 1.4 mg/dL of HDL. HDL Cholesterol levels have been found to be more sensitive to aerobic exercise than other lipid fractions [11]. The impact on HDL cholesterol may also differ by exercise type [12].

Besides exercise, diets have been associated with cholesterolemic profile [13,14]. Associations have been found between dietary interventions and lipid profile in children and adolescents [15]. Significant associations have been found between low-carbohydrate plant-based diets and other lipid fractions but not HDL cholesterol [16]. A review of 30 observational and 19 clinical studies found a 3.6 mg/dL and 3.4 mg/dL reduction in HDL cholesterol following a plant-based vegetarian diet [17]. A short-term very low carbohydrate diet has also been associated with increased HDL cholesterol in normal weight, normolipidemic women [18]. Based on our review of past literature, exercise would increase HDL-C levels while vegetarian diets would decrease it [19].

HDL-C has antioxidative, anti-inflammatory, and anti-thrombotic properties [20]. It has been demonstrated to be a protector factor for atherosclerosis although medical treatments that increase its concentration have failed, in randomized clinical trials, to show cardiovascular benefits. Therefore, increasing HDL by “conventional methods” such as exercise, non-smoking, and healthy diets are the only ways that might have an impact on cardiovascular health. A vegetarian diet is assumed to be healthier but there is scarce evidence on its actual effect on cardiovascular disease. To our knowledge, previous studies have not focused on the combined effect of diet and exercise. In this light, we assessed the impact of vegetarian diets and exercise on HDL cholesterol levels among an adult Taiwanese population.

## 2. Material and Methods

### 2.1. Data Source

Clinical and lifestyle data between 2008 and 2015 were obtained from Taiwan Biobank, a national resource with genetic information of Taiwanese adults aged 30–70 years. Recruitment into the biobank started in 2008 and is ongoing. Prior to recruitment in the biobank, all participants gave informed consent. The biobank data are separated into categories including questionnaires, physical examination, blood and urine tests, biological samples, and experimental data. The investigation conformed to the principles outlined in the Declaration of Helsinki. The Institutional Review Board of Chung Shan Medical University approved this study (approval number CS2-16114; approved on 14 September 2016).

### 2.2. Study Participants

Data from 9588 participants (5162 women and 4426 men) with no history of cancer were analyzed. The basic characteristics included sex, age, lifestyle factors (diet type, smoking, drinking and coffee drinking), biochemical information (total cholesterol; TC, triglycerides; TG, low-density lipoprotein; LDL-C with cut-point of 130 mg/dL [21], high-density lipoprotein; HDL-C) and anthropometric measures (waist-hip ratio; body mass index; BMI, and body fat, and WHR, with cut-points of <0.9 for men and <0.8 for women [22].

Details of dietary intake were collected using questionnaires contained in the biobank [23]. Participants were categorized as omnivores (people who consumed food of both plant and animal origin), former vegetarians (people who have adhered to a vegetarian diet for at least 6 months in their lifetime but who were no longer on vegetarian diet during recruitment into the biobank), lacto-ovo vegetarian (people that consumed eggs, milk, and dairy products) and strict vegetarian (people who consumed plant-based food). Vegetarians included people who avoided all animal flesh, including fish and poultry. Further stratifications were made by exercise levels, which were estimated from the physical activity questionnaire. Exercise was defined as physical activity at least 3 times a week lasting at least 30 min in duration [23,24]. Respondents answered ‘yes’ to having participated in at most three of the physical activity patterns listed (including jogging, strolling, rope jumping, swimming, gymnastics, yoga, taijiquan, qigong, Chinese martial arts, hiking, biking, badminton, table tennis, soccer, golf, tennis, basketball, other ball games, weight training, aerobic dance, ballroom dance, and hula hoop [25].

### 2.3. Statistical Analysis

The SAS 9.4 software (SAS Institute, Cary, NC, USA) was used for statistical analyses. A Chi-square test was used to compare HDL-C levels between different exercise groups. Associations of HDL cholesterol levels with diets were determined using multiple linear regression analysis. Data were presented as means ± standard error for continuous variables.

## 3. Results

**Figure 1:**
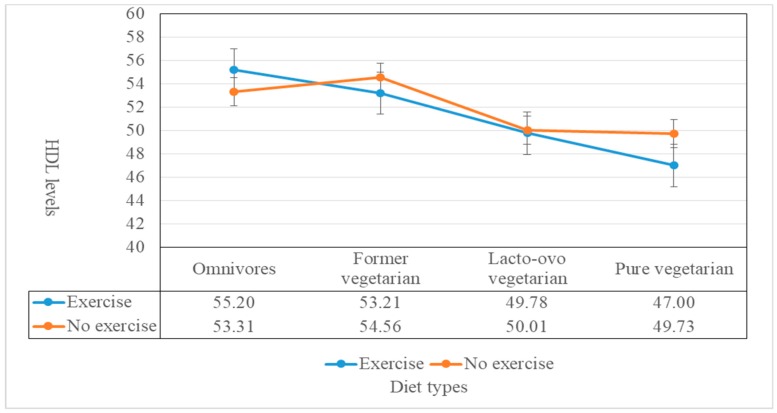
Mean HDL-C levels (mg/dL) according to diet group and exercise.

The final recruits included 4025 exercisers and 5563 non-exercisers (Table 1). Overall, male and female exercisers were associated with higher HDL-C compared to non-exercisers. Exercisers who were omnivores, former vegetarians, lacto-ovo vegetarians, and strict vegetarians had mean HDL-C (mean ± SE) levels of 55.2 ± 0.22 mg/dL, 53.21 ± 0.89 mg/dL, 49.78 ± 0.93 mg/dL, and 47 ± 3.05 mg/dL, respectively. Likewise, non-exercisers who were non-vegetarians, former vegetarians, lacto-ovo vegetarians, and strict vegetarians had mean HDL-C levels of 53.31 ± 0.19 mg/dL, 54.56 ± 0.75 mg/dL, 50.01 ± 0.71 mg/dL, and 49.73 ± 1.9 mg/dL. The highest level of HDL-C was found in exercisers and specifically among omnivores (55.2 mg/dL) while the lowest level was found in exercisers who were strict vegetarians (Table 1 and Figure 1).

Table 2 shows the association between diet type, exercise, and HDL. After adjusting for sex, age, TC, TG, LDL-C, WHR, BMI, body fat, smoking, drinking, and coffee intake, HDL-C levels were significantly higher in exercisers compared to non-exercisers. Compared with omnivores, strict vegetarians had lower levels of HDL-C (*β* = −5.705; *p* = 0.001) followed by the lacto-ovo vegetarians (*β* = −3.900; *p* < 0.001) and former vegetarians (*β* = −0.329; *p* = 0.475). The test for trend was significant (*p* < 0.001). After categorization based on exercise status, the decreasing trend in HDL among the different diet types was more striking in the exercise group (Table 3). The decrease was in a dose-response manner (*p* < 0.001). The *β* values were −13.984 (*p* < 0.001) for strict vegetarians, −4.419 (*p* < 0.001) for lacto-ovo vegetarians, and −1.864 (*p* = 0.019) for former vegetarians, respectively. Further analyses showed antagonistic interactions between diet and exercise (*p* = 0.009).

## 4. Discussion

To our knowledge, this study is the first to examine the combined effect of diets and exercise on HDL cholesterol levels in Taiwan. We found that vegetarian diets were associated with lower HDL-C levels. Similar results have been previously reported [17]. We also found that when compared with omnivorous diet, strict vegetarian diets were associated with lower HDL-C (*β* = −5.683) followed by Lacto-ovo vegetarian (*β* = −3.894) and former vegetarian (*β* = −0.306) diets. Results from a previous meta-analysis showed no differences between plasma HDL-C of vegetarians and omnivores [26]. However, the study is limited in that it was based on unadjusted estimates. In the current study, we adjusted for several variables. In another study, a vegetarian diet was associated with a 3.9 mg/dL decrease in HDL-C [27]. Another study investigating the impact of diet on lipid profile reported significant associations with other lipid fractions but not HDL-C [6].

Physical activity alone has a positive impact on HDL cholesterol [12]. Of the lipid fractions, HDL-C is reported to be the most sensitive to exercise [11]. However, we found that the addition of exercise led to greater reductions in HDL-C levels of strict vegetarians (*β* = −13.984) and lacto-ovo vegetarians (*β* = −4.419) compared with the omnivores. Only modest reductions were observed in former vegetarians (*β* = −1.864). Nonetheless, the test for trend was found to be significant. In their review, Ho and colleagues found that a combination of diet and exercise intervention led to greater improvements in HDL-C, but the difference became nonsignificant at one year of follow-up [15]. However, we found in the current study that regular exercise in conjunction with strict vegetarian diets were associated with greater reductions in HDL cholesterol levels. Increased HDL-C has been linked to a running distance in vegetarians [19]. However, the diverse forms of vegetarian diets were not considered. While assessing HDL-C levels, previous studies have mostly considered exercise and diets separately. In the current study, it was necessary to combine both variables considering their significant impact on metabolic risk factors associated with cardiovascular disease [15].

Physical inactivity and poor diet are responsible for heart disease, which is the second leading cause of death in Taiwan. Based on a 2015 survey conducted by the Health Promotion Administration (HPA), about 76% of Taiwanese individuals do not get sufficient exercise. However, efforts have been made to increase awareness of the benefits of exercise on health. Taiwan is one of the countries with the highest rate of vegetarianism. Such a plant-based culture has been cultivated by Buddhist vegetarian practices [28]. Despite this, the prevalence of heart disease in the Island remains relatively high. As mentioned above, there is evidence to show that plant-based diets have antioxidants and can protect against heart disease. Although a vegetarian diet has been associated with decreased HDL-C [29], exercising is considered to be an excellent way for vegetarians to boost their HDL-C levels [11]. Both the vegetable-rich diet and exercise training have been traditionally considered beneficial for cardiovascular health. However, based on the current study, a combination of these two factors appear detrimental. The underlying mechanism through which exercise plus a strict vegetarian diet would lower HDL-C remains to be clarified. Of note, a strict vegetarian diet does not contain dairy food and eggs, hence may not contain trans fatty acids and saturated fatty acids that can sufficiently raise HDL-C.

As stated above, HDL-C has been associated with heart diseases. However, causal associations between HDL-C and atherosclerotic cardiovascular disease (ASCVD) risk have been widely reported in epidemiologic but not Mendelian studies [30]. According to the 2019 European Society of Cardiology and European Atherosclerosis Society (ESC/EAS) Guidelines for the management of dyslipidemias, elevating levels of HDL-C do not reduce cardiovascular disease risk [30]. Nevertheless, updated dyslipidemia guidelines in Asia and the Middle East have suggested that HDL-C is important for improving risk estimates of ASCVD [2]. Of note, it is worth investigating HDL-C considering that dyslipidemia in Asia is mostly characterized by low-HDL-C as stated earlier. Moreover, region-wide guidelines for the management of dyslipidemia in Asia is yet to be fully established [2].

Despite our findings, the study limitations are worth mentioning. First, the information was based on self-report, hence we may not rule out the possibility of recall bias. Second, there was no information on the average daily nutrient intake. Besides, this study involved only a limited number of strict vegetarians who exercised regularly, so the results may have been influenced by individual variance. Finally, delineating between cardio-versus strength training and their impact on HDL-C would be valuable. However, our physical activity questionnaire did not have information on the appropriate intensity and energy expenditure measures.

## 5. Conclusions

Broadly speaking, we found that (1) HDL-C levels were higher in exercisers compared to non-exercisers. (2) Regular exercise in conjunction with strict vegetarian diets led to greater reductions in HDL cholesterol levels. Compared with omnivores, strict vegetarians were associated with lower HDL-C followed by lacto-ovo vegetarians. Based on these findings, regular exercise together with strict vegetarian diets might not serve as healthful behaviors to be implemented in everyday life considering the negative impact on HDL-C. We conclude that, while these results are significant, caution must be exercised. Stronger study designs are needed to understand whether associations observed are causal.

## Figures and Tables

**Table 1:** Mean HDL-C levels among study participants by exercise level and diet type.

Variables	No Exercise (n = 5563)		Exercise (n = 4025)		*p*-Value
	N	Mean HDL (SE)	N	Mean HDL (SE)	
Diet Type					0.0003
Omnivore	4927	53.31 (0.19)	3662	55.20 (0.22)	
Former vegetarian	353	54.56 (0.75)	191	53.21 (0.89)	
Lacto-ovo vegetarian	254	50.01 (0.71)	163	49.78 (0.93)	
Strict vegetarian	29	49.73 (1.90)	9	47.00 (3.05)	
Sex					0.0062
Female	3061	57.84 (0.24)	2101	59.36 (0.29)	
Male	2502	47.56 (0.22)	1924	49.97 (0.26)	
Age (years)					<0.0001
≤40	2051	53.90 (0.29)	526	55.11 (0.59)	
41–50	1713	53.08 (0.32)	884	55.13 (0.45)	
51–60	1238	52.87 (0.37)	1514	54.52 (0.34)	
>60	561	51.91 (0.52)	1101	55.03 (0.41)	
TC (mg/dl)					<0.0001
<200	3377	51.88 (0.21)	2228	52.55 (0.26)	
≥200	2186	55.28 (0.29)	1797	57.75 (0.33)	
TG (mg/dl)					0.0051
<150	4327	56.05 (0.19)	3226	57.36 (0.23)	
≥150	1236	43.31 (0.25)	799	44.82 (0.32)	
LDL-C (mg/dl)					0.0241
<130	3482	53.74 (0.24)	2428	54.95 (0.29)	
≥130	2081	52.34 (0.25)	1597	54.74 (0.30)	
WHR					0.1157
Male <0.9; Female <0.8	2150	54.91 (0.29)	1492	55.86 (0.36)	
Male ≥0.9; Female ≥0.8	3413	52.15 (0.22)	2533	54.29 (0.26)	
BMI (Kg/m^2^)					<0.0001
<18.5	179	65.42 (1.07)	71	68.37 (1.78)	
18.5 ≤ BMI < 24	2604	57.56 (0.25)	1971	58.68 (0.31)	
24 ≤ BMI < 27	1588	49.87 (0.29)	1264	52.21 (0.34)	
BMI ≥ 27	1192	46.37 (0.30)	719	47.77 (0.38)	
Body Fat (%)					<0.0001
Male <25; Female <30 (Ref)	2789	55.64 (0.26)	2250	56.21 (0.30)	
Male ≥25; Female ≥30	2774	50.78 (0.23)	1775	53.18 (0.30)	
Smoking habit					<0.0001
No	4260	54.89 (0.20)	3186	56.31 (0.24)	
Former	560	48.79 (0.48)	543	50.16 (0.51)	
Current	743	46.95 (0.43)	296	48.04 (0.70)	
Drinking					<0.0001
No	5021	53.48 (0.18)	3588	55.37 (0.23)	
Former	128	45.09 (0.94)	153	47.90 (0.86)	
Current	414	52.48 (0.67)	284	52.28 (0.74)	
Coffee drinking					0.2096
No	3743	52.57 (0.21)	2757	54.52 (0.25)	
Yes	1820	54.55 (0.32)	1268	55.63 (0.39)	

Data are expressed as means ± SE. TC indicates total cholesterol; TG, triglycerides; LDL, low-density lipoprotein; WHR, waist-hip ratio; BMI, body mass index.

**Table 2:** Association of HDL-C with diet type and associated factors.

Variable	*Β*-Coefficient	*p*-Value
Diet Type (Ref: Omnivore), n = 8589		
Former vegetarian, n = 544	−0.306	0.5064
Lacto-ovo vegetarian, n = 417	−3.894	<0.0001
Strict vegetarian, n = 38	−5.683	0.0008
P for trend		<0.0001
Exercise (Ref: no)		
Yes	1.146	<0.0001
Sex (Ref: female)		
Male	−7.841	<0.0001
Age (Ref: 30–40)		
41–50	0.144	0.6253
51–60	−0.318	0.3011
61–70	−0.185	0.6053
TC (Ref: <200)		
≥200	10.282	<0.0001
TG (Ref: <150)		
≥150	−10.168	<.0001
LDL-C (Ref: <130)		
≥130	−6.142	<0.0001
WHR (Ref: Normal)		
Abnormal	−2.281	<0.0001
BMI (Ref: 18.5 ≤ BMI < 24)		
<18.5	5.602	<0.0001
24 ≤ BMI < 27	−3.159	<0.0001
BMI ≥ 27	−4.469	<0.0001
Body Fat Rate (Ref: Normal)		
Abnormal	−1.758	<0.0001
Smoking habit (Ref: no)		
Former	−0.463	0.2094
Current	−2.051	<0.0001
Drinking (Ref: no)		
Former	−0.483	0.4569
Current	4.296	<0.0001
Coffee drinking (Ref: no)		
Yes	0.592	0.0099

Ref. = reference group.

**Table 3:** Association of HDL with diet type based on physical exercise.

Variables	No Exercise (n = 5563)		Exercise (n = 4025)	
	*β*-Coefficient	*p*-Value	*β*-Coefficient	*p*-Value
Diet Type (Ref: Omnivore), n = 8589				
Former vegetarian, n = 544	0.549	0.3263	−1.864	0.0194
Lacto-ovo vegetarian, n = 417	−3.563	<0.0001	−4.419	<0.0001
Strict vegetarian, n = 38	−3.134	0.0963	−13.984	<0.0001
P for trend		−		<0.0001
Sex (Ref: female)				
Male	−7.840	<0.0001	−7.960	<0.0001
Age (Ref: 30–40)				
41–50	0.341	0.3129	−0.594	0.3204
51–60	0.216	0.5708	−1.297	0.0208
61–70	−0.558	0.2643	−0.646	0.2771
TC (Ref: <200)				
≥200	10.022	<0.0001	10.625	<0.0001
TG (Ref: <150)				
≥150	−9.750	<0.0001	−10.798	<0.0001
LDL-C (Ref: <130)				
≥130	−5.711	<0.0001	−6.691	<0.0001
WHR (Ref: Ref: Normal)				
Abnormal	−2.233	<0.0001	−2.437	<0.0001
BMI (Ref: 18.5 ≤ BMI < 24)				
<18.5	5.016	<0.0001	7.234	<0.0001
24 ≤ BMI < 27	−3.450	<0.0001	−2.738	<0.0001
BMI ≥ 27	−4.532	<0.0001	−4.344	<0.0001
Body Fat Rate (Ref: Normal)				
Abnormal	−1.833	<0.0001	−1.710	0.0001
Smoking habit (Ref: no)				
Former	−0.647	0.1910	−0.123	0.8263
Current	−2.375	<0.0001	−1.646	0.0196
Drinking (Ref: no)				
Former	−0.088	0.9253	−0.826	0.3658
Current	5.406	<0.0001	2.6732	0.0001
Coffee drinking (Ref: no)				
Yes	0.636	0.0302	0.529	0.1508

Diet * exercise (*p* < 0.0001). Ref. = reference group.
